# Salivary Gland Secretory Carcinoma in the Facial Region: A Pediatric Case Presentation

**DOI:** 10.7759/cureus.98466

**Published:** 2025-12-04

**Authors:** Alexandra Perales, Miguel Flores, Carlos Manresa, Mariana Villarroel-Dorrego

**Affiliations:** 1 Oral and Maxillofacial Surgery Service, Hospital General del Oeste Dr. José Gregorio Hernández, Caracas, VEN; 2 Oral Pathology, Oral Medicine, Oral Surgery Department, Universidad Santa María, Caracas, VEN; 3 Dental Research Institute, Universidad Central de Venezuela, Caracas, VEN

**Keywords:** mammary analogue secretory carcinoma, parotid gland, pediatric patient, salivary gland neoplasm, salivary glands secretory carcinoma

## Abstract

Salivary gland secretory carcinoma (SC) represents a rare, slow-growing, low-grade malignant neoplasm that occurs mainly in the parotid gland. It has a slight predilection for the male gender and usually appears in adulthood. The morphologic and immunohistochemical features are like those observed in secretory breast carcinoma; however, it has been described as a distinct salivary gland tumor. Its clinical characteristics remain nonspecific and therefore can be misdiagnosed. This report describes a 12-year-old patient who presented with a slow, progressive, painless, and deforming mass at the level of the left lower third of the face, of eight months' evolution. After clinical and imaging evaluation, an excisional biopsy was performed. The sample was sent for histopathological study, which reported salivary gland SC. Although this condition typically presents in adults, it can occur in children, and it is important to consider it among the neoplasms that can occur at this age.

## Introduction

Salivary gland secretory carcinoma (SC), previously known as mammary analogue secretory carcinoma, is a rare, low-grade malignant neoplasm of the salivary glands that occurs primarily in the parotid gland, followed by the submandibular gland, minor salivary gland, and accessory parotid gland. Its morphological and immunohistochemical similarities are similar to those observed in secretory breast carcinoma. Its characteristic clinical manifestation is the presence of a slow-growing, painless tumor lesion; however, these features remain nonspecific, and therefore it can be misdiagnosed [1-3]. Before being first described by Skálová et al. in 2010 [4], SC was histologically misdiagnosed as an acinar cell carcinoma. Both entities have similarities, but with different histological characteristics and outcomes. Furthermore, SC is characterized by the ETV6-NTRK3 fusion resulting from a chromosomal translocation [3-5].

SC has a low overall incidence, representing less than 0.3% of all salivary gland tumors. Commonly, they may be present in adulthood with slight male predominance, and there have been few cases reported in pediatric patients [6-10]. SC is an indolent malignant salivary gland neoplasm that rarely metastasizes and therefore has a good prognosis. However, its rarity in childhood makes it a diagnostic challenge. The use of immunohistochemical markers, such as S100, SOX10, and mammaglobin, is very helpful. While the demonstration of ETV6 rearrangement by FISH, RNA sequencing, or PCR would be ideal, it is not possible in all cases [3-5]. We describe an unusual case of SC in the facial region of a pediatric patient to highlight pediatric diagnostic pitfalls and management considerations.

## Case presentation

**Figure 1:**
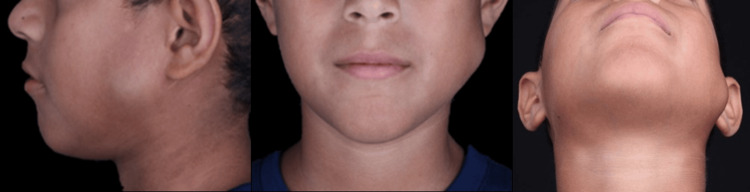
Extraoral photographs. Nodular, mobile lesion is observed in the left parotid-masseteric region.

A 12-year-old male patient was referred to the Department of Oral and Maxillofacial Surgery of the Hospital General del Oeste "Dr. José Gregorio Hernández," Caracas, Venezuela, with an extraoral normochromic and normothermic painless mass with slow growth on the left parotid-masseteric region evolving over eight months. It was mobile and soft to palpation (Figure 1). The patient had not received prior treatment for the lesion, and his mother did not provide any relevant medical history.

**Figure 2:**
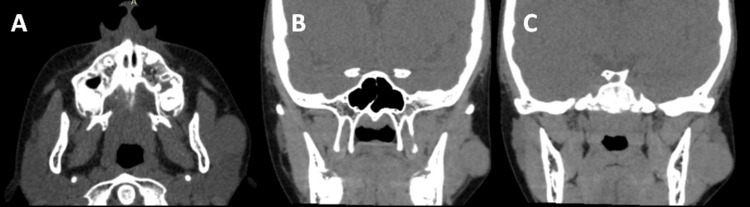
CT scan showing an isodense image corresponding to the lesion. Isodense image overlying the left parotid gland and masseter muscle and underlying cutaneous tissue, of 2.56 cm in larger diameter, with well-defined margins throughout its extent, corresponding to a tumor lesion. Axial (a) and coronal view (b and c).

Clinically, a nodular, mobile lesion was observed, separated from the muscular planes, soft to palpation, and completely asymptomatic. No adenopathy was found. A facial computed tomography revealed a 2.6 cm well-defined, encapsulated, isodense image, located in the left parotid-masseteric region, underlying subcutaneous tissue and superficial to the masseter muscle (Figure 2).

**Figure 3:**
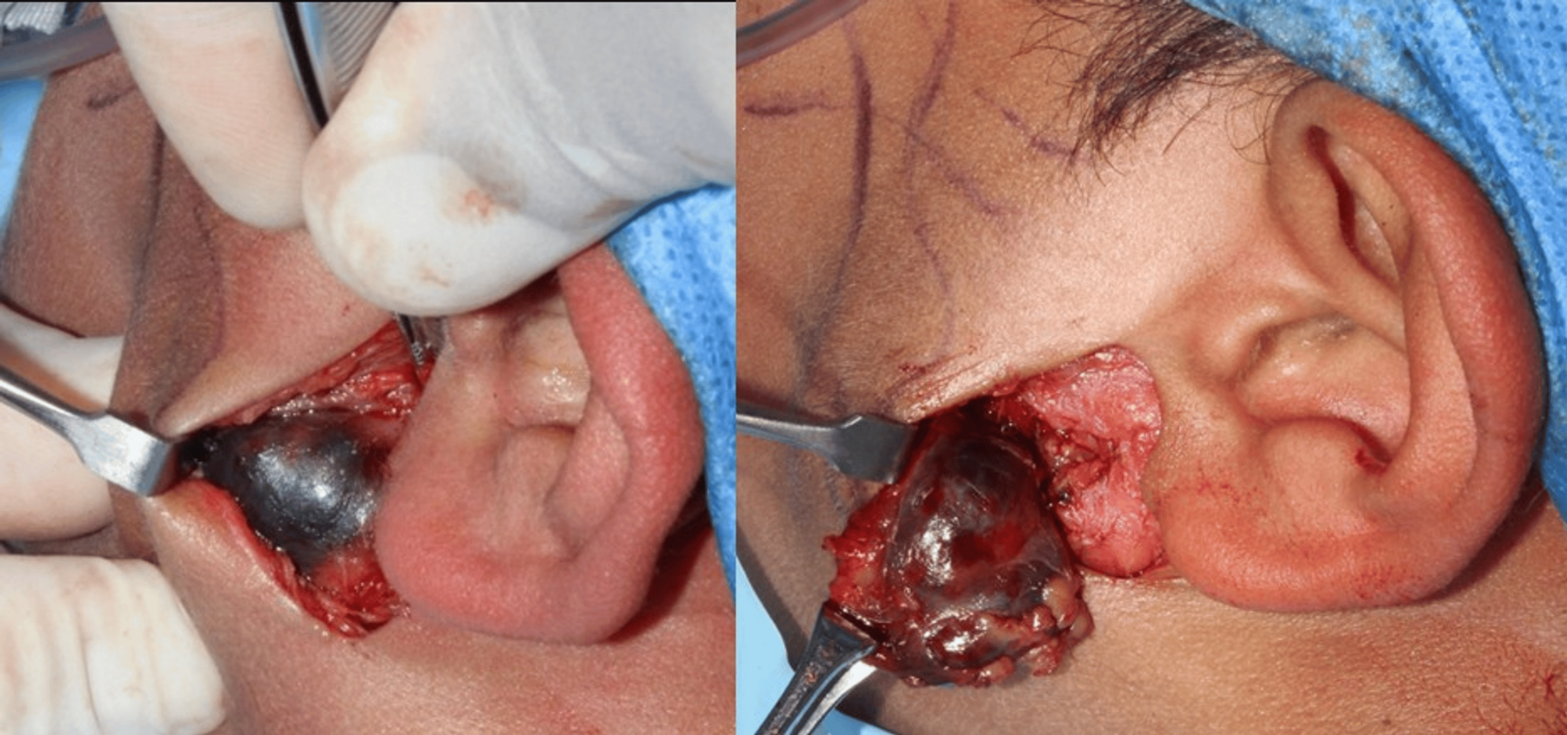
Excisional biopsy of the lesion. Preauricular surgical approach preserving nerve. Note the dark color of the tumor.

The clinical diagnoses proposed were dermoid cyst and pleomorphic adenoma, given the benign clinical characteristics of the lesion. Under intravenous conscious sedation, an excisional biopsy of the lesion was performed, preserving the facial nerve (Figure 3). After antiseptic and asepsis measures, markings were made for the preauricular approach. Local anesthetic (lidocaine 2% and epinephrine 1:100,000) was infiltrated, then an incision was made with a number 15 scalpel in the cutaneous and subcutaneous plane, and a blunt dissection was made until the lesion was located and excised. Hemostasis was controlled, and tissue synthesis was carried out layer by layer, completing the surgical act without complications.

**Figure 4:**
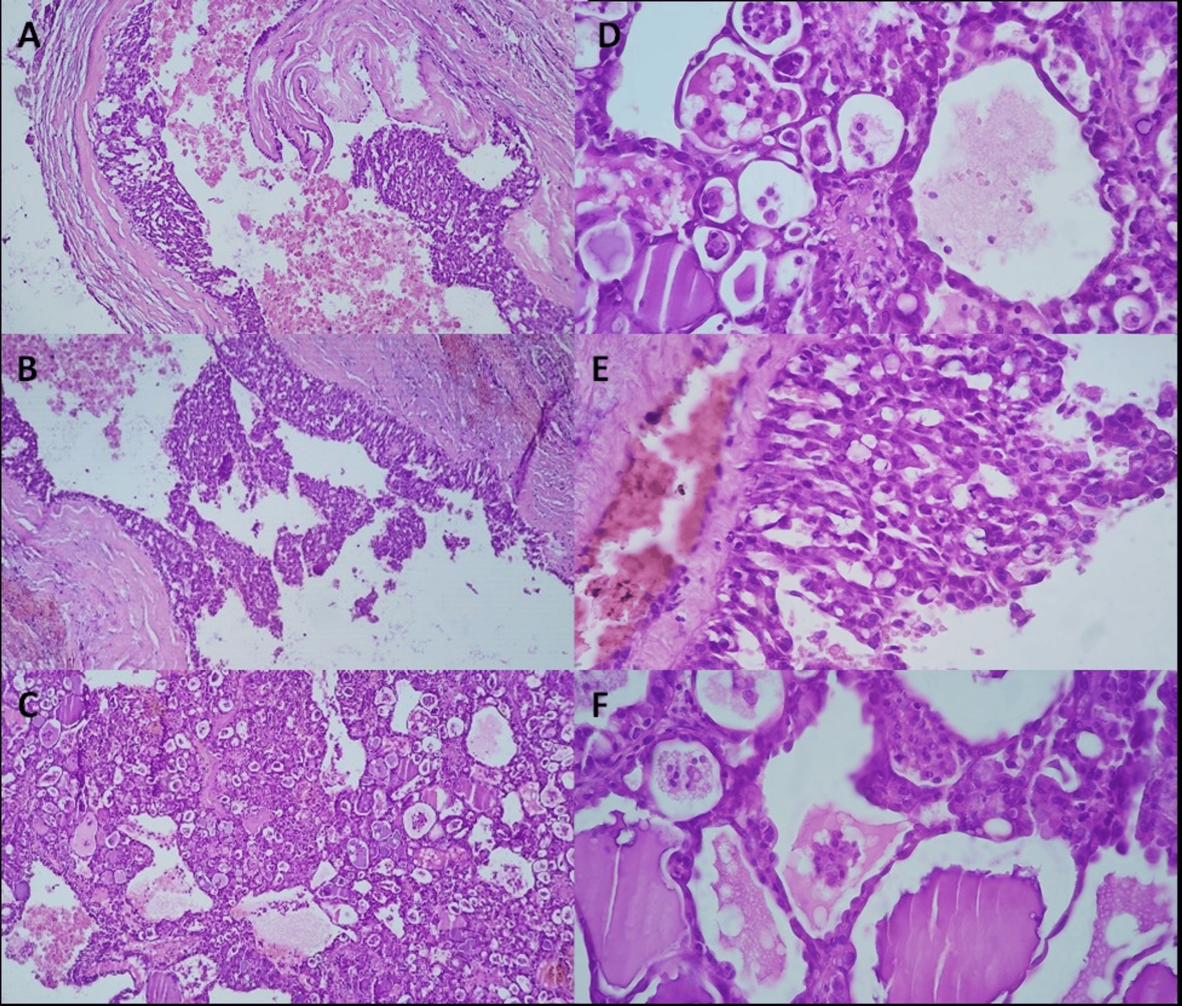
Histopathology sections with H&E staining Neoplasm showed  a well-circumscribed, non-encapsulated, glandular neoplasm,  with a macro and microcystic structures (4X H&E) (a-c). The lumen is outlined by rounded cells with round vesicular nuclei, fine granular chromatin, and prominent nucleoli. No perineural or vascular invasion was observed (10X H&E) (d-f). H&E: hematoxylin and eosin

A violet-colored, well-defined specimen was studied. Microscopic evaluation showed a well-circumscribed but not encapsulated glandular neoplasm. The tumor was composed of microcystic structures with distinctive luminal material (Figure 4). Completed excision with free margins was observed.

**Figure 5:**
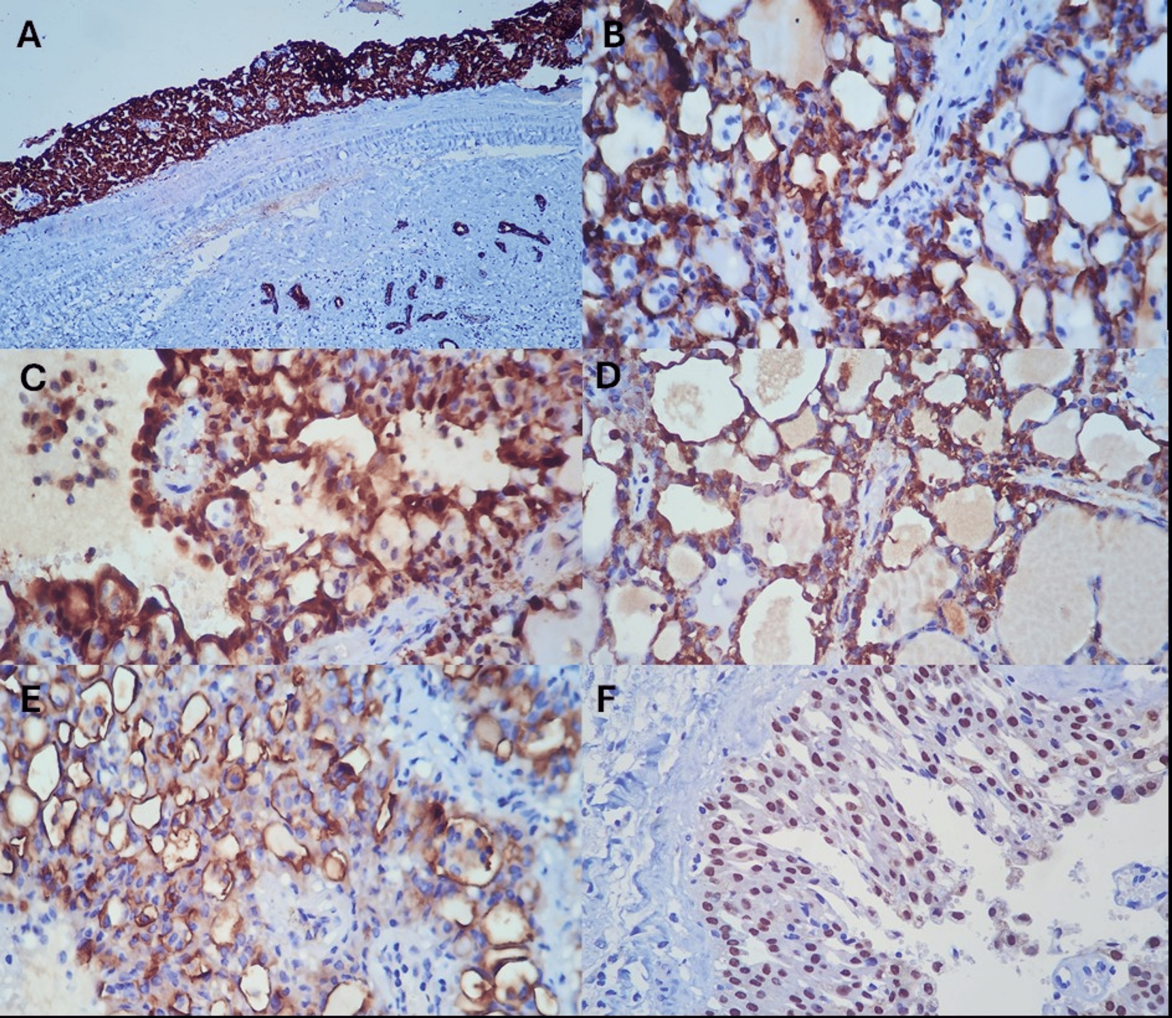
Immunohistochemistry staining. Expression of CK-7 (a,b), S-100 (c), mammaglobin (d), MUC4 (e), and SOX10 (f).

An immunohistochemistry study was performed, showing positivity for CK-7, S-100, mammaglobin, MUC 4, and SOX10 (Figure 5), confirming the diagnosis of SC. Molecular conformation of ETV6 or RET rearrangement was not carried out. 

The patient was referred to the pediatric oncology department, and after a clinical and paraclinical evaluation, the absence of disease was found. Additional therapy was not indicated; however, strict outpatient check-ups were established. Around 20 months of clinical follow-up have shown no evidence of neoplasia, and the sensory and motor function of the area remains intact.

## Discussion

SC represents a rare tumor of salivary glands in children. It was initially described by Skálová et al. in a case series of 16 salivary gland tumors that exhibited histomorphological and immunohistochemical characteristics remarkably similar to mammary secretory carcinoma [5].

SC usually presents as a low-grade, malignant lesion with a low rate of regional lymph node metastases and a low mortality rate. It has often been misdiagnosed as acinic cell carcinoma; therefore, it represents one of the main differential diagnoses, along with mucoepidermoid carcinoma and adenocarcinoma. However, the immunohistochemical and molecular profile of SC makes it possible to differentiate it from other salivary gland tumors [11-13].

The most notable characteristic in the present case is the patient's age, which does not coincide with the average age reported for this neoplasm, which is typically the fourth and fifth decades of life [9-14]. Though SC can occur in children, it is relatively rare. Cases reported in children and adolescents are summarized in Table 1. 

**Table 1:** Pediatric SC reported in the literature Cases reported in the literature as pediatric series or single cases of SC.

Author, year	Gender/Age (in years)	Location	Histopathology	IHC	Treatment
Meng et al., 2023 [8]	M, 4 y	Parotid gland	Solid, tubular and papillary growth patterns, eosinophilic secretions within microcystic structures	CK7, S100, and pan-TRK protein. Protein p63 was positive with a peripheral pattern	Excision with free margins
Ngouajio et al., 2017 [9]	M, 14 y	Parotid gland	Cystic, tubular, and/or papillary architecture	S100, mammaglobin, cytokeratin 19, and vimentin	Left superficial parotidectomy with selective neck dissection
Ash et al., 2023 [15]	M, 13 y	Parotid gland	Solid, microcystic, macrocystic, papillary-cystic, and tubular patterns	CK7 and S-100	Excision
Rastatter et al. 2012 [16]	F, 14 y	Parotid gland	Prominent papillary formations and several microscopic cystic spaces	S100	Parotidectomy
Hwang et al., 2014 [17]	F, 13 y	Parotid gland	Microcysts, tubular structures, and solid nests, with focal papillary formation	S100, CK 19, and vimentin	Excisional biopsy with free margins
Keisling et al., 2014 [18]	F, 5 y	Buccal mucosa	Microcystic spaces, foamy cells, and vacuolated cells	Vimentin, AE1/AE3, CK7. EMA, S100, and mammaglobin	Excisional biopsy
Joshi et al., 2015 [19]	M, 15 y	Parotid gland	Microcystic with containing vacuoles	S100, CK7, CK19, vimentin, EMA	Parotidectomy
Oza et al., 2016 [20]	F, 9 y; F, 16 y	Parotid gland	Papillary and microcystic pattern	Mammoglobin and S100	Parotidectomy
Chen et al., 2018 [21]	F, 12 y	Parotid gland	Microcystic pattern	CK7, S100, GATA3	Superficial parotidectomy
Shigeta et al., 2018 [22]	F, 7 y	Parotid gland	Microcystic structure	S100, mammaglobin, GCDFP15, vimentin, CAM5.2	Superficial parotidectomy
Shukla et al., 2018 [23]	M, 17 y	Parotid gland	Solid, cystic, tubular, and papillary architecture with individual cells having abundant eosinophilic granular to vacuolated cytoplasm	CK, EMA, S100 and mammaglobin	Excision of injury with free edges
de Melo et al., 2025 [24]	F, 9 y	Upper lip	Microcystic nests filled with eosinophilic secretions	CK7, mammaglobin, and S100. PAS positive	Excisional biopsy
de Souza Tolentino et al., 2025 [25]	F, 14 y	Palate	Tumor cells arranged in a microcyst architecture	-	Excisional biopsy, bilateral tonsillectomy, and adjuvant radiotherapy
Serrano-Meneses et al., 2024 [26]	M, 11 y	Left cheek	Cystic tumor, well-circumscribed and encapsulated. Intracystic cellular proliferation of monotonous cells with eosinophilic cytoplasm, round nuclei	CK (AE1-3), CK 7, GATA3, and S100. PAS positive	Resection
Moreddu et al., 2023 [27]	M, 8 y; F, 12 y	Parotid gland	Encapsulated, micro-cystic proliferation of monomorphic tumor cells with abundant eosinophilic cytoplasm	S100 and SOX10	Complete parotid excision surgery with neck node level II dissection
Woo et al., 2014 [28]	M, 14 y	Parotid gland	Microcystic, tubular, and solid growth patterns, as well as granular and vacuolated amphophilic cytoplasm	Vimentin, S100 and EGFR	Parotidectomy
Inaba et al., 2015 [29]	F, 15 y	Parotid gland	Microcystic pattern	S100 and mammaglobin	Parotidectomy
Quattlebaum et al., 2015 [30]	F, 15 y	Parotid gland	Dominant cyst with intracystic fragments of solid and papillary tan tissue with surrounding atrophic salivary gland tissue	S100 and CK19	Superficial parotidectomy with facial nerve dissection
Salgado et al., 2021 [31]	Four cases total: M, 7 y; M, 9 y; F, 14 y (n=2)	3 cases, parotid gland. 1 case, submandibular gland.	Well-circumscribed lesions composed of mid-size, monotonous cells with eosinophilic and sometimes vacuolated cytoplasm. Duct-like structures and microcysts with colloid-like material	S100, CK7, mammaglobin and GATA3	Excision of injury with free edges
Cardoni et al., 2023 [32]	F, 12 y	Maxillary sinus	Low-grade component: Papillary/tubular architecture, with cells with dense chromatin and inconspicuous nucleoli. High-grade component: solid pattern	CK (AE1/AE3), vimentin, CK7, EMA, S100 and SOX10, GATA3 and MUC4	Four courses of 5-fluorouracil and cisplatin plus oral TRK-inhibitor drug. Surgery for a suspected neoplastic residue in the ethmoid area. Adjuvant proton therapy on surgical bed (61.4 Gy) and on an area including the ipsilateral laterocervical lymph nodes (54.05 Gy).

Regarding the clinical presentation, studies agree that this is a painless, slow-growing, deforming tumor that can cause facial asymmetry. Occasional bleeding and ulceration of the adjacent mucosa have also been reported [11-12]. In terms of location, this entity has a predilection for major salivary glands, particularly the parotid gland [1], coinciding with the present case, where the lesion was encapsulated lateral to the superficial lobe of the left parotid gland and masseteric muscle.

Concerning the histopathological characteristics, almost all SC cases had typical microcystic architecture, bland tumor cells with eosinophilic to vacuolated cytoplasm, and abundant hypereosinophilic luminal secretion [13-14]. Other patterns include tubular, follicular, and papillary-cystic structures, and a few cases may show a macrocystic morphology. The tumour cells have low-grade vesicular round to oval nuclei with finely granular chromatin and distinctive, centrally located nucleoli. The pale pink cytoplasm is granular to vacuolated. Cellular atypia is usually mild, and mitoses are rare. High-grade transformation is uncommon. Very rare cases of high-grade show solid growth, tumor necrosis, and multiple mitotic figures. Immunohistochemical panel of S-100, mammaglobin, and DOG1 distinguishes SC from acinic cell carcinoma [13]. SC shows a diffuse and strong expression of pancytokeratins (AE1-AE3 and CAM 5.2), CK7, CK8, CK18, CK19, epithelial membrane antigen, S-100 protein, MUC4, SOX10 and mammaglobin [13-14].

Management of SC is based on oncological surgical resection. Lymphadenectomies, radiation, and chemotherapy its reserved for cases with regional involvement, histological risk factors (positive margins, perineural invasion), or metastatic disease. Yet, there is still no clear management established for the treatment of SC, due to the low numbers of cases and lack of evidence. Most SC cases involve low-grade malignant neoplasms with effective surgical management, which agrees with the patient reported in this work. Still, there are reports of high-grade, more aggressive cases with higher rates of recurrence [32]. Boon et al. [5] reported 31 patients with SC who underwent tumor resection. Only one patient developed a local recurrence, but no regional recurrences or distant metastases were observed. Furthermore, they found that overall survival at 5 and 10 years was 95% and disease-free survival was 89%.

## Conclusions

SC is an infrequent malignant neoplasm of the salivary glands that does not commonly affect children. Although rare, it can occur and mimic benign lesions due to its behavior and clinical characteristics. There are few reports of SC in the pediatric age, but many agree that the clinical course is indolent, which can lead to misdiagnosis. Clinicians should be aware of this possibility and include this diagnosis when encountering a pediatric patient. It should be noted that this is a case report of one patient, and it was not possible to perform genetic fusion testing, which is ideal for the diagnosis of SC.
